# Trends in the incidence and survival of multiple myeloma in South East England 1985-2004

**DOI:** 10.1186/1471-2407-10-74

**Published:** 2010-03-01

**Authors:** Christine Renshaw, Nicolas Ketley, Henrik Møller, Elizabeth A Davies

**Affiliations:** 1 King's College London, Thames Cancer Registry, 42 Weston Street, London, SE1 3QD, UK; 2 Queen Elizabeth Hospital NHS Trust, Department of Haematology, Stadium Road, Woolwich, London, SE18 4QH, UK

## Abstract

**Background:**

Multiple myeloma is an uncommon cancer with a poor prognosis. Its incidence is expected to increase due to ageing populations and better diagnosis, and new treatments have been developed to improve survival. Our objective was to investigate trends in the epidemiology and survival of multiple myeloma for South East England.

**Methods:**

Data on 15,010 patients diagnosed with multiple myeloma between 1985 and 2004 was extracted from the Thames Cancer Registry database. We calculated the yearly age-standardised incidence rates for males and females and age-specific incidence rates in 10-year age groups for both sexes combined. We also explored geographical variation in incidence across primary care trusts. We then used period analysis to calculate trends in 1- and 5-year relative survival over the 15 years 1990-2004, comparing survival by sex and by age group 59 years and below versus 60 years and above. Finally, we investigated 5-year relative survival for the period 2000-2004 by socio-economic deprivation, assigning patients to quintiles of deprivation using the Income Domain of the Index of Multiple Deprivation 2004 based on postcode of residence.

**Results:**

The incidence of multiple myeloma was higher in males than in females and in patients over 70, throughout the period 1985-2004. No obvious geographical pattern of incidence by primary care trust emerged. The 1- and 5-year relative survival of male and female patients increased in both age groups and was statistically significant in males aged over 60. There was a tendency for better survival in patients resident in the most affluent areas, but this did not reach statistical significance.

**Conclusions:**

The trends in incidence of multiple myeloma in males and females are similar to that reported from other western populations. Relative survival was higher for younger patients although we found significant improvements in 1-year relative survival for male patients over 60 years old. The improved survival demonstrated for patients of all ages is likely to reflect increased detection, earlier diagnosis and the introduction of new treatments. Future studies should investigate the influence of ethnicity on incidence and survival, and the effect of specific treatments on survival and quality of life.

## Background

Multiple myeloma is the second most common haematological cancer accounting for 10-15% of these malignancies [1]. The disease is a proliferation of malignant plasma cells within the bone marrow; these cells usually produce abnormal immunoglobulin-related proteins in the serum (paraprotein) and urine (Bence Jones protein). The disease is generally diffuse (hence "multiple" myeloma) but focal deposits are common, typically involving the spine, ribs, skull and pelvic bones. Patients present with non-specific symptoms such as fatigue, anaemia and bone pain, so many patients have advanced disease at the time of diagnosis [2]. The disease is essentially one that affects older adults (median age at presentation 70 years) and it is rare for it to be diagnosed in those under 40 years old [3].

Since multiple myeloma is a relatively rare disease its aetiology has been difficult to assess. Established risk factors include increasing age, male sex and a positive family history. In addition, incidence rates are consistently higher in black ethnic groups world-wide [4] and have recently been shown to be higher in black ethnic groups in England [5]. In 2004, the age-adjusted incidence of multiple myeloma in the UK was 5.8 per 100,000 standard European population for males and 3.8 per 100,000 population for females [6]. This rate is similar to that in Canada, Sweden and white Americans, [7] but higher than that in Asia populations [8]. Increases in incidence rates are also likely due to better diagnosis (immunohistochemistry and serum free light chain assays) and more accurate death certification. The number of cases is expected to increase in developed countries in line with the predicted increase of life expectancy of their populations [9].

Significant improvements in one-year relative survival have been observed since the 1970s [10,11] due to new treatments. A study by Phekoo and colleagues [12] developed a clinician-reported dataset for a population of 5.4 million part of South East England. They found that the incidence in 1999 to 2000 was slightly higher than previously reported in the UK, and survival was significantly reduced in patients older than 65 years old compared with younger patients. No marked association between socio-economic deprivation and patient survival was reported in England and Wales for the period 1986 to 1990 [10]. However, a recent analysis for 2000-2001 found that patients living in affluent areas have a 5-year relative survival of more than 10 percentage points higher compared to those living in the most deprived areas, and that the socio-economic difference in survival has widened between 1990 and 2000 [13].

In this study we used data from the Thames Cancer Registry area covering a population of 14.2 million living in South East England and report trends in the incidence and survival over a longer time period dating back to 1985.

We aimed to provide new information that would help guide clinical and health service planning to meet patient needs within the geographical area of South East England.

Our objectives were:

1) To describe trends in the incidence of multiple myeloma for men and women in South East England between 1985 and 2004.

2) To explore geographical variation in the incidence of multiple myeloma at the level of individual primary care trust between 1995 and 2004.

3) To describe trends in the relative survival for multiple myeloma for men and women for the period 1990 to 2004.

4) To investigate the relationship between relative survival and socio-economic deprivation of area of residence between 2000 and 2004.

## Methods

During the study period 1985 to 2004 the Thames Cancer Registry (TCR) covered the areas of London, Surrey, Sussex, Kent, Essex and Hertfordshire including a resident population of 14.2 million people. The population in London is ethnically diverse including 11% from Black ethnic groups, 12% from Asian ethnic groups and 71% from White groups, according to the 2001 Census. These proportions compare with those for the whole TCR area of South East England of 6% Black, 7% Asian and 83% White. In this area cancer registration is initiated by clinical and pathology information received from hospitals and by information on deaths provided by the National Health Service Central Register through the Office for National Statistics. Trained data collection officers collect further information on demographic details, disease stage and treatment received within the six months following diagnosis from the medical records of individual patients. In addition information from the South Thames Haematology Register was included from 1998, based on data supplied directly from clinical teams to the cancer registry. Data are checked in the registry to ensure that they refer to new malignancies rather than a recurrence of already registered cases, continuously added to a central database and quality assured. Information from death certificates of patients on the cancer register allows the calculation of survival after diagnosis.

Cancer registries in England have legal support to collect data relating to cancer under Section 251 of the NHS Act 2006 and formerly under Section 60 of the Health and Social Care Act 2001. The study used an anonymised dataset and separate ethical approval was not required.

We extracted data on 15,010 patients diagnosed with multiple myeloma (ICD-O M97323) between 1985 and 2004 from the registry database. We tabulated the demographic characteristics of these patients and calculated the age-standardised incidence rate for men and women for South East England using the European standard population. We used data on the size of the resident population to compute the age-specific incidence rates for multiple myeloma in each year for each of the age groups, less than 40, 40 to 49, 50 to 59, 60 to 69, 70 to 79 and 80 or older.

In the area there are 46 primary care trusts responsible for commissioning and funding health services for the needs of their local communities. We explored variation in the incidence across the population of primary care trusts (PCTs) by calculating the age-standardised incidence rates for each area, using the boundaries established by the NHS in October 2006. The PCT incidence rates were grouped into quintiles and displayed on maps, using geographic information system (GIS) software.

To determine the trend in survival over the period 1990 to 2004, we used period analysis to calculate the relative survival for multiple myeloma in men and women separately. The period analysis method [14] enables cross-sectional analysis by including all patients about whom there is information on the database during the follow-up period of interest, even if diagnosis occurred prior to this period. This enables the most recent data on patients diagnosed in the last years of the study period to be included in the analysis. The end point for the relative survival analysis in any period does not rely on the accurate classification of the cause of death. This provides a measure of total excess mortality associated with the diagnosis of multiple myeloma. Relative survival is the observed survival in the patient group divided by the expected survival of a comparable group from the population, in any given period, matched by age and sex. We grouped the patients into those diagnosed at ages 59 years and below and 60 years and over. We calculated relative survival estimates for the periods of follow-up 1990 to 1994, 1995 to 1999 and 2000 to 2004, excluding patients whose registrations were based only on their death certificate.

Finally, we investigated 5-year relative survival for the period 2000-2004 by socio-economic deprivation, assigning patients to quintiles of deprivation using the Income Domain of the Index of Multiple Deprivation 2004 [15] based on postcode of residence. The overall trend for the relative survival estimates was assessed by test for trend over socio-economic levels and over the three time periods.

## Results

Table 1 shows the demographic characteristics of patients with multiple myeloma diagnosed between 1985 and 2004 in South East England. Fifty-two percent of patients were male and 82% were older than 60 years. The median age at diagnosis was 72 years for this population.

**Table 1:** Characteristics of patients with multiple myeloma diagnosed between 1985 and 2004, in South East England

Characteristics	Number of patients	(%)
**Sex**		
Males	7733	(52)
Females	7277	(48)

**Total**	15010	(100)

		
**Age group**		
0-39	137	(1)
40-49	596	(4)
50-59	1854	(12)
60-69	3510	(23)
70-79	4989	(33)
80+	3924	(26)

**Total**	15010	(100)

Figures are numbers (with percentages in parenthesis) of patients.

**Figure 1:**
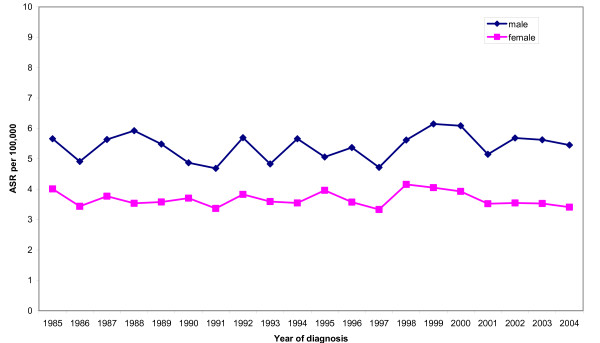
**Age-standardised incidence rates for multiple myeloma, South East England 1985-2004**.

Figure 1 shows the age-standardised incidence rates for males and females in South East England. Overall rates did not increase during the study period. Throughout the period 1985 to 2004 the age-standardised incidence rate in males was higher than for females.

**Figure 2:**
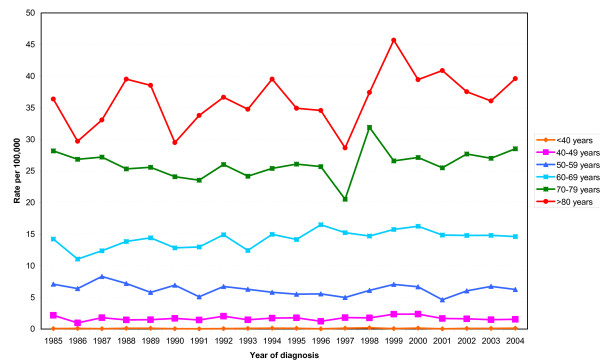
**Age specific incidence rates for multiple myeloma, male and female combined, South East England 1985-2004**.

**Figure 3:**
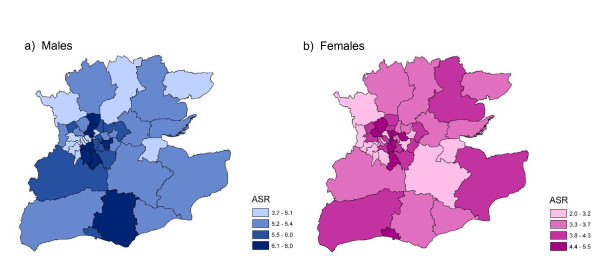
**Age-standardised incidence rates for multiple myeloma for primary care trusts in South East England, 1995-2004**.

Figures 2 shows the age specific incidence rates for males and females combined in different age groups. The highest rates were in the groups aged over 60 years. There was no obvious geographical patterning of incidence by primary care trust of residence (Figure 3). Higher incidence rates were found in the inner London PCTs but were not exclusive to these areas.

**Figure 4:**
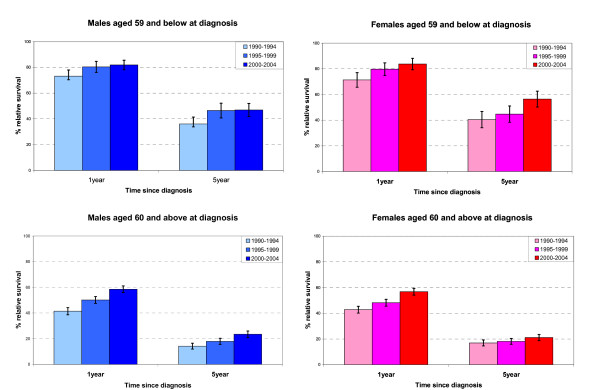
**Relative survival by period of follow-up for patients with myeloma in South East England 1990-2004**. 1- and 5-year estimates with 95% confidence intervals.

Relative survival was higher for the younger age group than for the older group in both males and females (Figures 4). There was also evidence of increasing relative survival over time for both sexes in each age group. Figure 4 shows the 1- and 5-year estimates of relative survival for patients aged 59 and below at diagnosis, by sex for the three follow-up periods. This group had a higher overall relative survival in all of the follow-up periods. For males the 5-year relative survival increased from 36% to 46% and 47%, with the largest improvement being between the 1990-1994 and 1995-1999 periods of follow-up. For females a steady increase was seen for each of the follow-up periods with 5-year relative survival yielding figures of 40% to 45% and 56%, respectively. Figure 4 shows the 1- and 5-year estimates of relative survival of patients aged 60 and above, by sex for the three follow-up periods. This older age group had a lower overall relative survival in all follow-up periods. For males the 5-year relative survival increased from 14% to 18% and 23%. Similarly in females, survival increased from 17% to 18% and 21% for each of the periods of follow-up. The 1-year estimates of relative survival showed a very similar pattern of improving survival over each of the three periods of follow-up, with a significant improvement in survival trend in the 1-year estimates in patients aged 60 and over. We also found an improvement in 5-year survival in males in the older age group over the time period.

**Figure 5:**
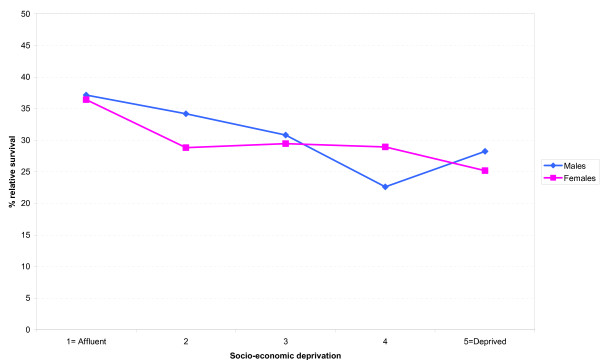
**Period analysis of 5-year relative survival by socio-economic deprivation, South East England 2000-2004**.

Figure 5 shows the relationship between 5-year relative survival and socio-economic deprivation for area of residence for the period 2000 to 2004. In both males and females there was a tendency for higher survival in patients resident in the most affluent areas, (males trend p = 0.09, females trend p = 0.07).

## Discussion

In this study using cancer registration data for South East England we found that the incidence of multiple myeloma was higher in males than in females over the period 1985 to 2004. The incidence rates increased sharply with age and were highest for men and women aged over 80, with relatively few cases occurring in patients less than 40 years old. Both findings are consistent with the well documented risk factors for multiple myeloma of advanced age and male sex. The overall incidence figures for South East England are also similar to those previously reported for the UK [6]. No obvious geographical pattern of incidence by primary care trust emerged, although higher rates in some inner London PCTs may be due in part to a higher proportion of people from Black ethnic groups resident in these areas.

Period analysis of relative survival demonstrated a trend for improvement in the survival of all patients between 1990 and 2004, with each successive five-year period showing improvement on the previous 1-year survival. Although this is generally consistent with the results of other recent large studies of similar-sized populations, our results differ in showing a significant improvement in 1-year survival in patients over 60 years old, though shorter than for those younger than 60. This may in part be due to the larger number of patients in the older age group. By comparison, a Swedish study [11] investigating relative survival over a 30-year period from 1973 to 2003 found that while 1-year survival had significantly improved for all age groups, improvements in 5-year survival were confined to patients younger than 70 years and in 10-year survival to those younger than 60 years. In the early years of the Swedish study younger patients will not have received autologous stem cell transplants, which has been the standard consolidation treatment for such patients since the 1990s. Improvements in the survival of younger patients are to be expected over this longer 30 year period. A recent North American study using a model-based projection method [16] found much larger increases in projected survival for the younger age group of below 45 years, than for the older patients above 60 years in whom projected increases were much lower and hardly exceeded the estimates from traditional survival analysis. A number of other studies have calculated survival using the Kaplan-Meier method, the most notable of which are an international study of 10,549 patients from North America, Europe and Japan [17] and another local study of 855 patients in the South Thames area [12]. Whilst a direct comparison of these results is not possible, these two studies confirm the improved survival for patients diagnosed at a younger age. Finally we found a tendency for better survival in patients resident in the most affluent areas, similar to Rachet and colleagues [13].

The treatment of myeloma has evolved dramatically over the study period, [18] yet our data and those of other population-based studies demonstrate that it takes many years for the promising results obtained from clinical trials on selected patients to translate into significant improvement of survival for a population of unselected patients. Autologous stem cell transplantation was first introduced in the mid-1980s and developed to become the standard consolidation therapy for younger patients over the next decade, [19] probably accounting for much of the improvement in relative survival described in patients 59 years and younger in our series.

The late 1990s saw the beginnings of "novel therapy" for myeloma with thalidomide, bortezomib and lenalidomide producing encouraging responses in relapsed and refractory cases [20]. These therapies are no longer novel in that we know that they can work; the challenge is to apply them to achieve improved survival on a population basis. As Schey argues, there is no reason why these agents should not improve outcome in elderly patients given their proven efficacy and tolerability in this age-group [21]. Our data begin to show an improvement in relative survival for those over 60, possibly a result of the wider and earlier application of thalidomide in particular across the UK.

A key focus of current research is maximizing complete response and remission in myeloma, which extrapolating from other haematological malignancies, may translate eventually into cure of this disease [22]. Thus it is important to note that not only 1-year relative survival but also 5-year survival are improved in both younger and older patients in our series. Continued improvement will require further clinical studies to explore how best to combine newer agents with conventional therapies, but will also require extension of the registry approach to ensure that the full impact of treatments on unselected patients is assessed and used to support sustained funding for these treatments. Integration of continuing clinical treatment data with traditional cancer registration methods will enable analysis of the specific interventions for improved survival. In addition, a significant proportion (11%) of the population of London is known to be of black ethnicity, (2001 Census) and it is now well established that incidence of multiple myeloma is higher in black ethnic groups [23,4,5]. Studies in the UK should investigate the relationship between ethnicity and survival.

### Limitations of the study

Figures for the incidence of multiple myeloma by PCT of residence must be interpreted with care as case ascertainment may have varied across them, particularly as the South Thames Haematology Register covered only part of the area during the study period. In addition London is the most ethnically diverse part of the UK, this study has not investigated the influence of ethnicity on incidence and survival. We reported no treatment analyses because only limited details of treatment received during the six months after diagnosis are collected for cancer registration purposes. Data on important new therapies and stem cell transplantation received after this time are therefore incomplete.

## Conclusions

The trends in incidence of multiple myeloma in males and females are similar to that reported from other western populations. No obvious geographical pattern of incidence by primary care trust emerged. Patients in affluent areas tended to have a better survival. Relative survival was longer for younger patients although we found significant improvements in survival for male patients over 60 years old. The improved survival demonstrated for patients of all ages is likely to reflect increased detection, earlier diagnosis and the introduction of new treatments.

## Competing interests

The authors declare that they have no competing interests.

## Authors' contributions

CR contributed to the study's conception and design, analysed the data and wrote the first draft of the paper. NK helped to design the study, interpreted the findings and write the paper. HM interpreted the findings and write the paper. ED contributed to the study's conception and design, interpreted the findings and manuscript writing. All authors read and approved the final manuscript.

## Pre-publication history

The pre-publication history for this paper can be accessed here:

http://www.biomedcentral.com/1471-2407/10/74/prepub
